# To each its own: Mechanisms of cross-talk between GPI biosynthesis and cAMP-PKA signaling in *Candida albicans versus Saccharomyces cerevisiae*

**DOI:** 10.1016/j.jbc.2024.107444

**Published:** 2024-06-04

**Authors:** Sneha Sudha Komath

**Affiliations:** Professor, School of Life Sciences, Jawaharlal Nehru University, New Delhi, India

**Keywords:** adenylyl cyclase, cAMP-PKA signaling, hyphae, pseudohyphae, GPI-*N*-acetylglucosaminyltransferase, Ras

## Abstract

*Candida albicans* is an opportunistic fungal pathogen that can switch between yeast and hyphal morphologies depending on the environmental cues it receives. The switch to hyphal form is crucial for the establishment of invasive infections. The hyphal form is also characterized by the cell surface expression of hyphae-specific proteins, many of which are GPI-anchored and important determinants of its virulence. The coordination between hyphal morphogenesis and the expression of GPI-anchored proteins is made possible by an interesting cross-talk between GPI biosynthesis and the cAMP-PKA signaling cascade in the fungus; a parallel interaction is not found in its human host. On the other hand, in the nonpathogenic yeast, *Saccharomyces cerevisiae*, GPI biosynthesis is shut down when filamentation is activated and *vice versa*. This too is achieved by a cross-talk between GPI biosynthesis and cAMP-PKA signaling. How are diametrically opposite effects obtained from the cross-talk between two reasonably well-conserved pathways present ubiquitously across eukarya? This Review attempts to provide a model to explain these differences. In order to do so, it first provides an overview of the two pathways for the interested reader, highlighting the similarities and differences that are observed in *C. albicans versus* the well-studied *S. cerevisiae* model, before going on to explain how the different mechanisms of regulation are effected. While commonalities enable the development of generalized theories, it is hoped that a more nuanced approach, that takes into consideration species-specific differences, will enable organism-specific understanding of these processes and contribute to the development of targeted therapies.

Glycosylphosphatidylinositol (GPI) biosynthesis is a reasonably well conserved pathway in all eukaryotes (for a general overview, see (1)). It generates a special kind of glycolipid which, when attached to the C-terminal end of proteins, marks them for transport to the extracellular leaflet of the plasma membrane (PM) and/or the cell wall (CW). Many kinds of GPI-anchored proteins adorn eukaryotic cell surfaces and enable them to interact with their external environment. They may range from enzymes, enzyme inhibitors, signal receptors, proteins required for nutrient uptake and complement regulators, to hydrolytic enzymes, host-recognition factors, adhesins, virulence factors, and proteins that coat the surface of pathogens to evade host immune response. Defects in GPI biosynthesis are lethal only at the embryonic stage in mammals, while in most lower eukaryotes, it affects viability (2). The GPI contains a conserved core in all eukaryotes, which is embellished with organism-specific, developmental stage-specific, or cell/tissue-specific modifications that customize it as per the specific requirements of the cell to which they belong (1). In some instances, these differences are achieved by altering the order of key biosynthetic steps. In other cases, they involve subtle differences in the active site geometry of specific enzymes, and in yet others, they are the outcome of a completely different set of regulatory mechanisms. Thus, despite the underlying structural conservation, differences abound, the possibility of using GPIs and GPI biosynthetic steps as vaccines and drug targets in eukaryotic pathogens continues to generate much interest (3, 4, 5, 6, 7).

This review, therefore, begins with an overview of the GPI biosynthetic pathway in the opportunistic human fungal pathogen, *Candida albicans*, relative to the much better elucidated pathway in the nonpathogenic model yeast, *Saccharomyces cerevisiae*. Both organisms appear to generate very similar mature GPI anchors containing four compulsory mannose residues in the carbohydrate core (8, 9). In mammals, a fourth mannose in the GPI occurs only in a tissue-specific manner (10). As will be described in greater detail in this review, the GPI biosynthetic steps as well as the corresponding enzymes identified in *S. cerevisiae* have homologs in *C. albicans* and much of the machinery involved in packaging and transport of GPI-anchored proteins to the cell surface is conserved. But *C. albicans* produces nearly twice the number of GPI anchored proteins as *S. cerevisiae*, many of which have no *S. cerevisiae* counterpart and are primarily required for the virulence/pathogenesis of the organism (11). What is now becoming increasingly clear is that the pathways are regulated very differently in the two organisms. One such regulation involves a role for the cAMP–PKA pathway (12, 13, 14).

In general, the cAMP–PKA pathway enables eukaryotic cells to respond to the signals it receives from the external environment. It does so by processing multiple cues *via* a central signal-recognizing hub, the adenylyl cyclase. These signals either directly activate the adenylyl cyclase or are recognized by other proteins which then stimulate the adenylyl cyclase to produce the second messenger, cAMP. This in turn activates PKA and the downstream signaling cascade. Subtle differences in the signal binding sites and/or in their receptors along with differences in the downstream targets ensure that the cAMP–PKA pathway is tailored for organism-specific functions. For example, cAMP in mammals is produced in response to cues such as growth factors, hormones, or cytokines, and abnormal activation results in disease (15). On the other hand, abundant glucose activates cAMP production in *S. cerevisiae* (16). High cAMP levels promote filamentation in *S. cerevisiae* and suppress it in *Saccharomyces bayanus* (16). In *Schizosaccharomyces pombe*, the cAMP–PKA pathway is required for sexual differentiation, but it is induced upon glucose starvation rather than by its abundance (17). In *Candida auris*, the cAMP–PKA pathway plays a role in filamentation and in virulence, but its PKA can be activated even in an adenylyl cyclase–independent manner (18).

Glucose induces cAMP-PKA–dependent filamentation in *C. albicans* just as it does in *S. cerevisiae* (19). But filamentation in *C. albicans* is also a major virulence trait (19). It is induced inside the human host by physiological temperature (37 °C), as well as by the presence of serum, glucose, amino acids, alkaline pH, and CO_2_, among other cues. Superficial infections by *C. albicans* appear to mostly involve yeast cells, while invasive infections appear to require the filamentous forms (19). All three cellular morphologies (yeast, pseudohyphae, and hyphae) are observed in disseminated candidiasis. However, cells locked in either morphologies and unable to transition between them are attenuated in virulence in bloodstream infection models (20, 21).

Combining hyphal morphogenesis with expression of hyphae-specific GPI-anchored virulence factors would contribute to the success of the organism as a pathogen. Indeed, the cAMP-PKA–dependent hyphal morphogenesis pathway is co-activated with GPI biosynthesis in this pathogen, and many GPI-anchored proteins are exclusively expressed in the hyphal form (11, 22). On the other hand, in *S. cerevisiae*, activation of filamentation is accompanied by a downregulation of the GPI biosynthetic pathway and *vice versa* (12).

The second part of this review, therefore, provides a discussion on the cAMP-PKA–dependent filamentation pathways in *S. cerevisiae* and *C. albicans* and the many points of divergence between them. The third and final section of the review attempts to synthesize the information provided in the preceding sections to propose a model on how and why diametrically opposite mechanisms of cross-talk exist between GPI biosynthesis and filamentation in *C. albicans versus S. cerevisiae* and discuss what its implications could be.

## GPI biosynthesis and GPI anchored proteins in *S. cerevisiae* and *C. albicans*

### The GPI biosynthetic pathway

GPI synthesis begins on the cytoplasmic face of the endoplasmic reticulum (ER) with the transfer of GlcNAc from UDP-GlcNAc to phosphatidylinositol (PI) and is completed in the ER lumen to produce ethanolamine-PO_4_-6(Manα1-2)Manα1-2Manα1-6Manα1-4GlcNα1-6(acyl)myo-inositol-1-PO_4_-lipid in *S. cerevisiae* and *C. albicans*. Once generated in a roughly linear set of enzyme-catalyzed reactions, the complete precursor GPI is attached to the C-terminal ends of proteins possessing a GPI attachment signal sequence. Remodeling of their GPI anchors and ER-exit of GPI-anchored proteins occur in another series of sequential steps, with each preceding step promoting the next. They are then transported through the secretory pathway, *via* the Golgi, to the PM and the CW. These three broad processes are briefly elaborated below.

### Formation of the complete precursor and its attachment to proteins

**Figure 1:**
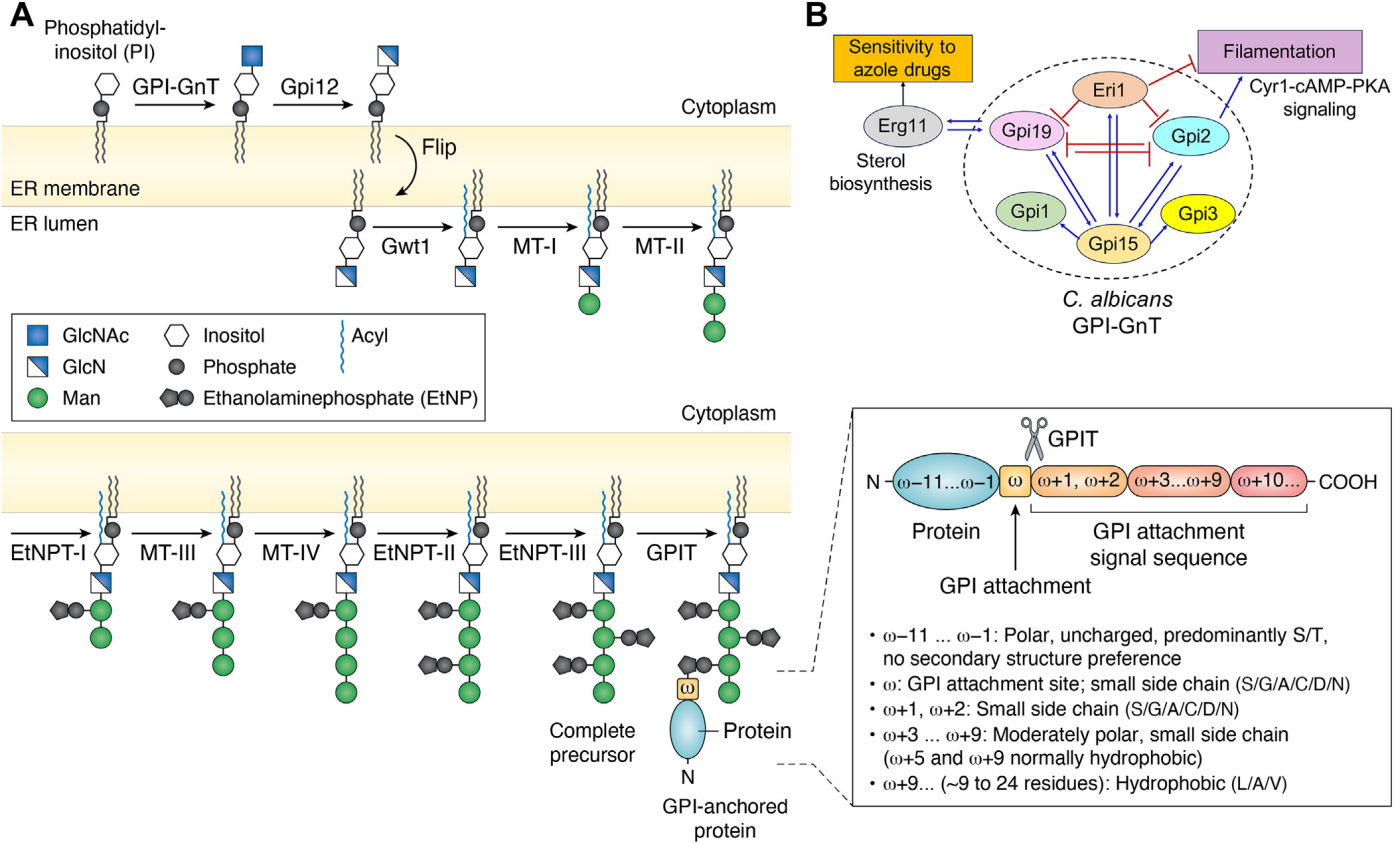
**The GPI biosynthetic pathway.***A*, formation of the complete GPI precursor and its attachment to proteins. The pathway shown here is from what is known in *Saccharomyces cerevisiae*. Not all steps have been elucidated in *Candida albicans*. However, homologs of most of the enzymes and their subunits are also present in *C. albicans* and the final GPI product is similar. GPI biosynthesis begins at the cytoplasmic face of the ER when GlcNAc from UDP-GlcNAc is transferred to PI by the action of a GlcNAc transferase (GPI-GnT). GlcNAc-PI is de-*N*-acetylated by the action of Gpi12 and then flipped into the ER lumen by the action of an unknown flippase. The first step on the lumenal face involves acylation (palmitoyl) by an acyltransferase, Gwt1. Subsequently four Man residues and three EtNP residues are transferred by the action of different mannosyltransferases (MT-I to MT-IV) and EtNP transferases (EtNPT-I to EtNPT-III), respectively, to generate the complete precursor (CP). The donor for the mannoses is Dol-P-Man and for EtNP is ethanolaminephosphate. The most likely sequential pathway alone is shown. Once CP is produced, it is amide linked to the C-terminal end of proteins by the action of a transamidase (GPIT). For this, GPIT cleaves the C-terminal GPI attachment signal sequence (SS) of proteins and attaches the EtNP on Man-3 of the CP to the newly generated carboxylate terminus. Inset: The nature of the GPI attachment signal sequence is conserved. The amino acids of the GPI attachment signal sequence are not conserved, but their nature is. These common features, mentioned in the figure, make it easy to identify them either manually or using bioinformatic tools. GPI attachment occurs at the ω residue. *B*, the known intra-subunit transcriptional cross talk within the *C. albicans* GPI-GnT and cross-talk with other biochemical pathways in the cell. Intersubunit transcriptional regulations within the GPI-GnT are unique to *C. albicans* and have not been reported in any other organism. Activations are represented by *black arrows* within the dotted circle representing the enzyme complex and the *red flatheaded arrows* represent repression within the GPI-GnT. Gpi15 activates the expression of both Gpi2 and Gpi19 while Eri1 represses both. Gpi15 and Eri1 mutually activate one another. Gpi2 and Gpi19 mutually inhibit the expression of one another and independently activate the expression of Gpi15. The roles of other GPI-GnT subunits in this schema are not yet elucidated. Cross-talk with other biochemical pathways is observed at this step. CaGpi19 and Erg11 (in sterol biosynthetic pathway) are mutually transcriptionally activated. Since Erg11 is the target of azoles, this affects the response of the cells to azole antifungals. CaGpi2 and CaEri1, in different ways, control filamentation *via* the Cyr1-cAMP-PKA pathway. CaGpi2 activates cAMP-PKA signaling for filamentation while CaEri1 represses it. The *red flatheaded arrows* represent inhibition and the *blue arrows* represent activation.

The transfer of GlcNAc from UDP-GlcNAc to PI, to produce GlcNAc-PI, is the first step of the GPI biosynthetic pathway (Fig. 1*A*). It involves a rather unique UDP-GlcNAc:PIα1-6*N*-acetylglucosaminyltransferase, referred to as GPI-GnT from here on. Unlike most glycosyltransferases found elsewhere in the cell, the enzyme involved in the GPI biosynthetic pathway is a multi-subunit membrane-bound complex, referred to as GPI-GnT from here on. It has six subunits, Gpi1, Gpi2, Gpi3, Gpi15, Gpi19, and Eri1, common to both *S. cerevisiae* and *C. albicans* (see Ref. (23) and the references therein). A seventh subunit (Yil102c-A), which stimulates GPI-GnT activity in *S. cerevisiae* and is a mammalian DPM2 homolog (24). Although a DPM2 homolog is annotated in the *C. albicans* genome database, it has not yet been identified as a GPI-GnT subunit in *C. albicans*.

Gpi3 is predicted to be the catalytic subunit of the GPI-GnT, based on its homology with bacterial transferases and on its ability to be photocrosslinked to a substrate analogue, P(3)-(4-azidoanilido)-uridine 5′-triphosphate [-^32^P] (25). CaGpi3 is predicted to be a homolog of Gpi3 based on sequence similarity (26). Yet, jawsamycin, a natural product produced by *Saccharomyces luteoverticillatus*, is able to inhibit Gpi3 from *S. cerevisiae* and fungi of the Mucorales order but not CaGpi3, suggesting significant differences in their active site geometries (7). Indeed CaGpi3 does not complement a conditional null *gpi3* strain (unpublished data, Komath lab).

The exact roles of other subunits in catalysis by the GPI-GnT are not yet worked out. But several lines of evidence point to unique regulatory roles for at least some of them in controlling the GPI biosynthetic pathway. Neither CaEri1 (unpublished data, Komath lab) nor CaGpi2 (27) are functional homologs of their *S. cerevisiae* counterparts within the GPI-GnT. A deletion mutant of *CaGPI19* lacking codons for 1 to 150 N-terminal amino acids complements a conditional null strain of *S. cerevisiae GPI19* (28) while the full length *CaGPI15* complements a conditional null strain of *S. cerevisiae GPI15* (13).

Not only are the subunits of the GPI-GnT in *C. albicans* different from that of *S. cerevisiae*, but there is also a unique transcriptional cross-talk between them which is entirely absent in *S. cerevisiae* (13, 14, 22, 29, 30, 31). *CaGPI15* appears to be a master regulator controlling the expression of both *CaGPI2* and *CaGPI19* while *CaERI1* is their common repressor (Fig. 1*B*). *CaGPI15* and *CaERI1* are mutual activators of one another. A mutually negative regulation exists between *CaGPI2* and *CaGPI19* and both can independently activate *CaGPI15*. The participation of other GPI-GnT subunits in this cross-talk is yet to be investigated. Cross-talk with other biochemical pathways is also observed at this step. *CaGPI19* appears to control sensitivity to azole drugs *via* mutually activating transcriptional cross-talk with *ERG11,* a crucial gene in the sterol biosynthetic pathway that encodes lanosterol 14-α-demethylase, the target of azoles. How CaGpi2 and CaEri1 control filamentation *via* the Cyr1-cAMP-PKA pathway is a point we will return to in the last section of this review.

GlcNAc-PI formed at the end of Step-I above, is de-*N*-acetylated by Gpi12, a GlcNAc-PI de-*N*-acetylase. Due to organism-specific differences at this step, Gpi12 homologs in protozoan parasites have been investigated as drug targets (32, 33). CaGpi12 is a functional homolog of *S. cerevisiae* Gpi12 (34). This step is followed by the flipping of GlcN-PI into the ER lumen by an as yet unidentified mechanism. That a flippase might be involved has been suggested from assays using rat liver ER proteoliposomes (35), but a positive identification of a likely candidate is yet to happen. Some studies have suggested that Arv1 might be the elusive GPI flippase (36, 37).

Once flipped inside the lumen, the inositol ring of GlcN-PI receives an acyl (palmitoyl) chain at 2-OH by the action of an inositol acylase, Gwt1 (38). In general, fungal enzymes are distinct from the human enzyme and specific inhibitors of homologs from *Neurospora crassa,* *Cryptococcus neoformans*, several *Candida* sp., *Aspergillus* spp.*, Fusarium* spp. and *Scedosporium* sp., are reported (39, 40). Hence, as mentioned in the introduction, this step has been the intense focus of research for the development of antifungals and fungicides (5, 6, 41).

The acylated GPI intermediate stays trapped within the ER lumen for the remainder of the biosynthetic steps. It receives two mannose (Man) residues sequentially, one by the catalytic action of mannosyltransferase-I and the next by the action of mannosyltransferase-II. The former comprises two subunits, Gpi14 and Pbn1 in *S. cerevisiae* while the latter comprises subunits Gpi18 and Pga1 (42, 43, 44, 45, 46). CaGpi14 is a functional homolog of Gpi14 (47). Mcd4 adds a ethanolaminephosphate (EtNP) to Man-1 of the GPI intermediate formed at the end of either of these steps (48, 49). It too has been of interest as an antifungal drug target (50). A third crucial Man (Man-3) is added to the GPI intermediate by the action of Gpi10 (51). The action of Smp3 adds a fourth Man residue (Man-4), which appears to be necessary for Gpi13 to add a EtNP on Man-3 (8, 9). A final EtNP is then added on Man-2 by Gpi7 to generate the final complete precursor, Manα1-2(EtNP)Manα1-2(EtNP)Manα1-6(EtNP)Manα1-4GlcN-(acyl)PI (Fig. 1*A*) (52, 53). Dolichol-phosphate-mannose is the donor of the Man residues in all cases and phosphatidylethanolamine the donor of EtNP.

The complete GPI precursor is attached to the C-terminal end of proteins that carry an appropriate GPI attachment signal sequence with specific features (Fig. 1*A* (Inset)) (54). The enzyme that catalyzes this process needs to possess endopeptidase activity to remove the signal sequence as well as to catalyze a transamidation reaction which will link the newly formed carboxyl end of the protein to the free amine of the EtNP present on Man-3 of the complete precursor. The enzyme that does this is called the GPI transamidase (GPIT). It is composed of five subunits Gpi8, Gpi16, Gpi17, Gaa1 and Gab1 in *S. cerevisiae*, all of which appear to be essential for viability (55). Gpi8/CaGpi8 are likely to be cysteine protease-like endopeptidases. Indeed CaGpi8 is able to cleave a fluorescently labeled peptide substrate (56). The amide bond is hypothesized to be formed by Gaa1, a protein that classifies as a member of the M28 family of aminopeptidases (57, 58). The roles of the other subunits are perhaps structural. Phenotypes of GPIT mutants of *C. albicans* suggest a possible cross-talk of this step too with other cellular pathways (23), although this needs more in-depth investigations. Tentatively, however, it may be possible to suggest that such cross-talk at the first and last biosynthetic steps enables GPI production to be regulated in response to other biochemical changes in the cell.

### Remodeling of the GPI anchor within the ER lumen

**Figure 2:**
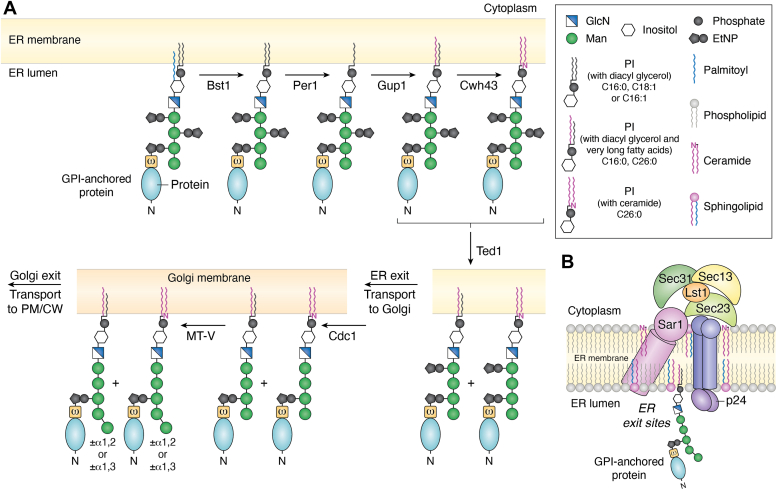
**GPI remodeling and ER exit of GPI-anchored proteins using COPII vesicles.***A*, remodeling of the GPI anchor: The GPI anchor attached to proteins are remodeled in the ER lumen before they are transported out. Bst1 first deacylates the inositol. Per1 then removes the short chain fatty acid at sn2 position to produce a lyso-PI. Next, Gup1 attaches a very long chain fatty acid at this position. In a fraction of GPI-APs, the diacylglycerol may then be entirely replaced by a ceramide *via* the action of Cwh43. Finally, the EtNP on Man-2 is removed by Ted1. This step is crucial for recognition of the GPI-AP by the p24 proteins and their ER exit *via* COPII-coated vesicles. *B*, recruitment of p24 and COPII proteins for ER exit: In the lumen of the ER, GPI-APs containing remodeled very long chain lipid tails and lacking the EtNP on Man-2 accumulate at the ER exit sites. Here, they are recognized and bound by the p24 family of four membrane proteins. Once the cargo is bound, the cytoplasmic domains of the p24 proteins recruit Lst1 and Sec23, forming a pre-budding complex, which is also promoted by the association of Sar1, a membrane-bound GTPase, within this complex. The outer coat proteins, Sec13 and Sec31, can now assemble upon the pre-budding complex causing a resultant bending of the membrane and formation of nascent vesicles which are pinched off by the catalytic activity of Sar1.

Once the protein is attached to the glycolipid anchor, many embellishments to the glycolipid core that were added at the start of the biosynthetic process begin to be removed or replaced with other moieties (Fig. 2*A*). The first to go is the acyl chain, which is removed by Bst1, an inositol deacylase (59, 60). This is followed by remodeling of the lipid chains of the PI. The C18:1 (or C16:1) fatty acid at the *sn2* position is removed by Per1, and a C26:0 chain is added at that position by Gup1 (61, 62, 63). Frequently, at this point in the biochemical pathway, the lipid chains of the PI are completely replaced with a ceramide by the action of Cwh43 (64, 65). Indeed a substantial fraction of fungal GPI-anchored proteins possess a ceramide lipid. Finally, the removal of EtNP from Man-2 by Ted1 enables GPI-anchored proteins to exit the ER (66, 67). *C. albicans* Per1, Cwh43 and Ted1 have not yet been analysed in a similar fashion.

### ER exit and transportation to the PM and CW

GPI-anchored proteins with their remodelled lipid chains accumulate at sphingolipid-rich ER exit sites for transport (Fig. 2*B*). This sequestering requires the very long C26:0 ceramides synthesized in the ER membrane as well as the C26:0 fatty acid chains of the GPI anchored protein (68, 69). Here they encounter the cargo receptor complex comprising four membrane proteins of the p24 family (Emp24, Erv25, Erp1, Erp2) which specifically recognizes the remodeled glycan cores of GPI-anchored proteins, lacking EtNP on Man-2 (67, 70). Once the cargo is bound, the cytoplasmic tails of the p24 proteins recruit Lst1 and Sec23 to form a pre-budding complex, which in turn help to assemble the outer coat proteins, Sec31 and Sec13, and form the nascent vesicles for GPI transport (68). These vesicles bud-out from the ER by the action of Sar1, a GTPase, and travel to the Golgi where the anchor and the protein are further modified. Homologs to each of these proteins exits in *C. albicans* as well and much of the machinery for ER exit and transport appears conserved (71).

The GPI loses an EtNP from Man-1 by the action of Cdc1 in the cis and medial faces of the Golgi (72). Here, approximately 5% of GPI-anchored proteins also receive a fifth Man α1,3 linked onto Man-4, while another ∼15% receive a Man α1,2 in the trans-Golgi instead (73). The protein itself may also be extensively glycosylated within the Golgi. How the GPI-anchored proteins exit the trans-Golgi, is not very well understood. In *S. cerevisiae* Gas1 seems to be transported to the PM with the help of a small GTPase, Arl1, while others such as Cwp1 and Crh1 or Crh2 are not (74). While this would argue against a general mechanism for the transport of GPI-anchored proteins, it is unclear what other mechanisms of transport might operate. Further, it remains to be seen whether these differences are attributable to differences in their lipid anchors (diacylglycerides *versus* ceramide lipids) or whether there are protein-specific specialized mechanisms involved.

Some GPI-anchored proteins remain localized to the PM while many have their lipid tails cleaved by glycosidases which act on the bond between Man-1 and GlcN. The choice of proteins undergoing this process might depend on specific sequences within the protein or its GPI attachment signal sequence, or might be dictated by differences in their lipid tails (66, 75, 76). It is also possible that a combination of these factors is involved. The proteins lacking the lipid anchor are then crosslinked to CW β-glucans *via* Man-1. Two putative GPI-anchored transglycosidases, Dcw1 and Dfg5, are proposed to play such a role in *S. cerevisiae* (77). Homologs of both enzymes exist in *C. albicans* as well as other fungi (78).

### GPI-anchored proteins in *C. albicans* and *S. cerevisiae*

Many different kinds of proteins may be GPI anchored in *S. cerevisiae* (79). They may be involved in CW integrity as structural proteins cross-linked to the β-glucans or may participate as enzymes such as glucanases, hydrolases, transglycosidases, chitinases, and even proteases. Some directly participate in CW biogenesis and remodeling, and some in flocculation, or budding, or cell separation, or mating. Thus, they may be expressed at specific time points in the cell cycle or to perform a specialized function. Many of these have counterparts in *C. albicans* as well. However, many GPI-anchored proteins are organism specific (80), and are likely to have evolved for organism-specific functions. For example, the Als family of eight adhesins required for adherence to the host by *C. albicans* has no homologue in *S. cerevisiae*. Similarly, the Cu/Zn dependent superoxide dismutases are exclusive to *C. albicans*. They enable the pathogen to survive oxidative stress conditions within the host and are important determinants of its virulence. In general, the number of GPI-anchored proteins in *C. albicans* are roughly twice that of *S. cerevisiae* (11). Interestingly, several of these are proteins that are expressed exclusively in the hyphal form of *C. albicans* and play a role in the success of the organism as a human pathogen (11, 81).

## Filamentation in *S. cerevisiae* and *C. albicans*

Filaments of *S. cerevisiae* are not true hyphae. They are pseudohyphal filaments, where daughter cells bud and separate from the mother cells but remain attached to the latter *via* a septum (16). When grown on solid media, many strains exhibit invasive growth, penetrating into the solid agar medium on which they are grown. Several signals and overlapping signaling pathways regulate these morphological transformations (82). The four major ones in *S. cerevisiae* are

i. the cAMP dependent protein kinase A (PKA) pathway,
ii. the filamentation activating mitogen activated protein kinase (fMAPK) pathway,
iii. the target of rapamycin (TOR) pathway, and
iv. the AMP-activated protein kinase (AMPK) sucrose non-fermenting 1 (Snf1) pathway.

Glucose or nitrogen limitation turns on the fMAPK pathway and activates Kss1, a downstream Ser/Thr MAPK (83, 84). The TOR pathway, as the name suggests, is inhibited by rapamycin and induced by nitrogen sources (85). The cAMP/PKA pathway and the AMPK/Snf1 pathway are both regulated by glucose (86, 87). But while the former is activated by it, the latter is repressed by glucose and other fermentable sugars and is activated when glucose is depleted. Intersections and cross-talk between these pathways and with other signaling networks in the cell are well established. For example, fMAPK is only one of at least three intersecting MAPK pathways, the other two being the pheromone-responsive mating MAPK and the osmotic stress responsive high-osmolarity glycerol 1 (Hog1) pathways (84). Similarly, Ras2 feeds into the fMAPK as well as the cAMP/PKA pathway (88).

In *C. albicans*, round or ovoid yeast cells are the most common ones observed during planktonic growth at 30 °C. Ellipsoid pseudohyphal cells where the daughter cell remains attached to the mother cell at distinct septa, and filamentous hyphal cells where these septa are lost, can both be induced from the yeast cells under appropriate conditions. ‘Filaments’ or ‘filamentation’ are used as umbrella terms in this Review to represent both morphogenetic states, since they are often simultaneously induced. Multiple pathways coordinate filament formation in this pathogen (19, 89). The four major ones are

i. the cAMP/PKA pathway
ii. the Cek-MAPK pathway
iii. the HOG-MAPK pathway, and
iv. the Rim101 pH-responsive pathway.

The cAMP/PKA pathway is activated, among others, by stimuli such as glucose, lactose, *N*-acetylglucosamine (GlcNAc), CO_2_, 37 °C temperature, peptidoglycans, serum, and amino acids (90). Cell cycle arrest in the G1 phase can also trigger filamentation *via* this pathway (91). The Cek-MAPK pathway responds to CW damage, low nitrogen or matrix embedding conditions (92, 93). The HOG-MAPK pathway responds primarily to osmotic or oxidative stress, and the Rim101 pH-responsive pathway turns on hyphal specific genes in response to alkaline pH (94, 95). Filamentation in *C. albicans* may also be repressed by the Nrg1/Tup1 and Rfg1/Tup1 mediated pathways (96, 97). As in *S. cerevisiae*, activation of multiple pathways by the same signal and cross-talk between different pathways leads to a complex network of interactions controlling hyphal development in *C. albicans* (89, 90). Feedback regulation of filamentation *via* quorum sensing molecules is reported in both *C. albicans* as well as in *S. cerevisiae* (98, 99, 100, 101).

In general, filamentation induced by cAMP-PKA remains the most prominent and extensively studied of morphogenetic signaling pathways in both organisms. For the sake of this Review, the discussion that follows will focus on this signaling cascade and its activation by Ras or Ras-independent processes, due to the manner in which it regulates and is regulated by the GPI biosynthetic pathway. But first, a description of the individual components of the pathway and how they come together to regulate filamentation.

### The cAMP-PKA signaling in *S. cerevisiae* and *C. albicans*

**Figure 3:**
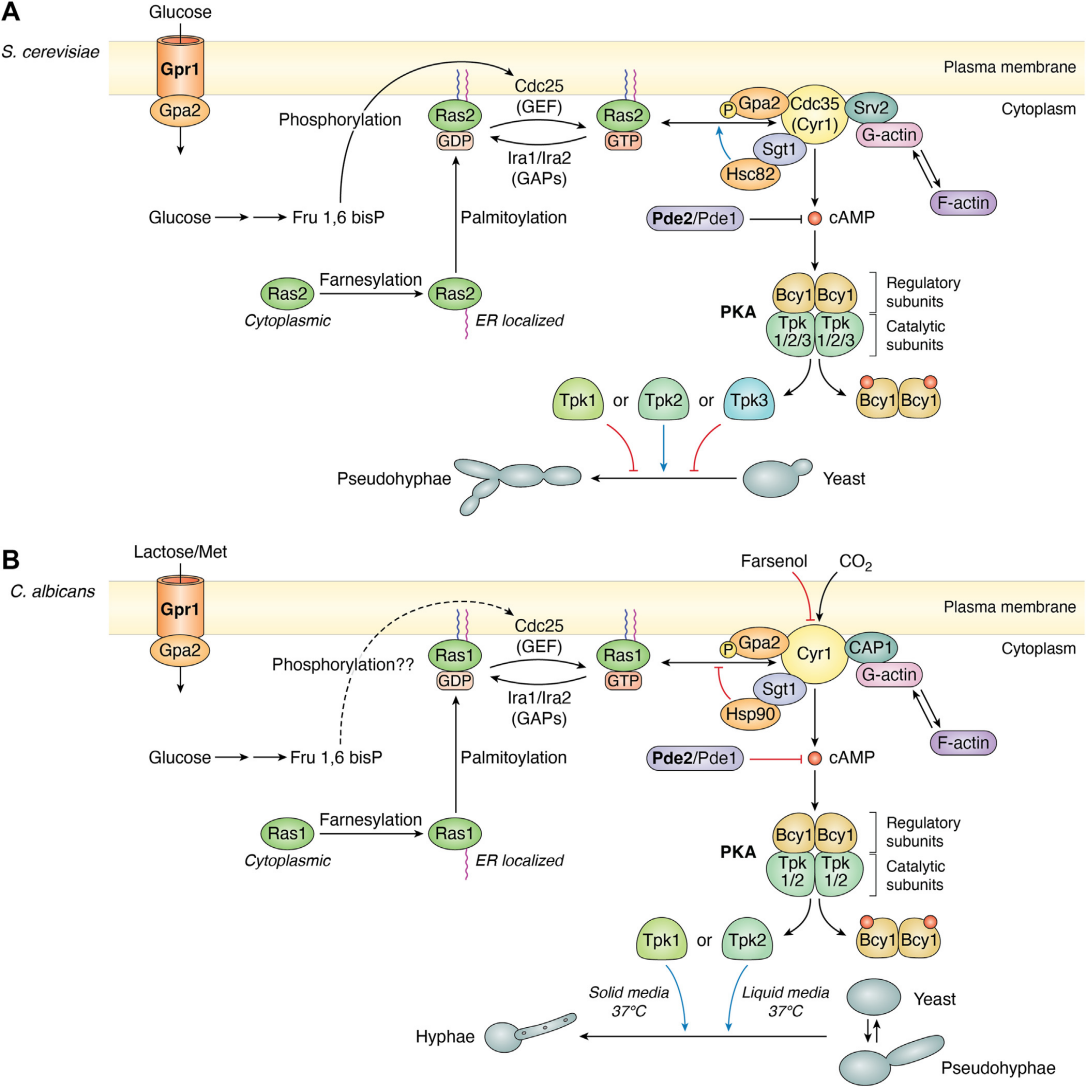
**The cAMP–PKA pathway that controls filamentation in *Saccharomyces cerevisiae* and *Candida albicans*.** A schematic representation of the pathway in (*A*), *S. cerevisiae* and (*B*) *C. albicans*. PKA is a complex of the form R_2_C_2_ which activates filamentation. The R subunits are constituted by Bcy1 while the C subunits may be any two of Tpk1/Tpk2/Tpk3 in *S. cerevisiae* and Tpk1/Tpk2 in *C. albicans*. When cAMP is produced, it binds to Bcy1 and releases the active C subunits which phosphorylate downstream transcription factors and activate them. Depending on the C subunits released, the downstream targets vary. The production of cAMP is made possible by the adenylyl cyclase (Cyr1) which coverts ATP to cAMP. Any excess cAMP is degraded by phosphodiesterases (Pde2/Pde1). Pde2 (in *bold*) is the dominant player. Cyr1 is regulated by the binding of Ras2/Ras1 in *S. cerevisiae* and by Ras1 in *C. albicans*. It is also activated by Srv2/CAP1 and the binding of monomeric G-actin to the latter. In *S. cerevisiae*, heat shock proteins Hsc82 (or Hsp82) along with the co-chaperone, Sgt1, promote the interaction between Ras2 and Cyr1, whereas in *C. albicans*, Hsp90-Sgt1 inhibits Ras1–Cyr1 interaction. Ras proteins require to be in their active GTP-bound form for an effective interaction with Cyr1. For this, the GDP-bound inactive form of Ras must first be activated by the GEF, Cdc25 to the GTP-bound form. Ras cycles back to its inactive state by interacting with GAPs, Ira1/Ira2 in *S. cerevisiae* and Ira2 in *C. albicans*. This interaction can be blocked by a Gly19Val mutation in *S. cerevisiae* Ras2 and Gly13Val mutation in *C. albicans* Ras1, producing constitutively activated Ras proteins. PM localization of Ras proteins is dependent on their farnesylation in the cytoplasm and palmitoylation at the ER. Gpr1-Gpa2 is a G-protein–coupled receptor-G-protein pair required to transport glucose (*S. cerevisiae*) or lactose/Met (*C. albicans*). Phosphorylated Gpa2 can activate Cyr1. The conversion of glucose to fructose 1,6-bis-phosphate (Fru1,6bisP) activates Cdc25 and turns on Ras-dependent Cyr1-cAMP-PKA signaling in *S. cerevisiae*. It is hypothesized that a similar mechanism operates in *C. albicans*. CO_2_-mediated activation of Cyr1 is exclusive to *C. albicans* and occurs independent of Ras1–Cyr1 interaction. *Double headed arrows* represent protein–protein interactions, the *red flatheaded arrows* represent inhibition, and the *blue arrows* represent activation.

The cAMP-PKA pathways of *S. cerevisiae* and *C. albicans* show an overall degree of conservation. An overview of the cAMP-PKA signaling pathway in *S. cerevisiae* and *C. albicans* is given in Figure 3.

#### PKA and its downstream targets

Phosphorylation of downstream targets by PKA can control filamentation in *S. cerevisiae* and *C. albicans*. PKA is a heterotetrameric enzyme made of two regulatory (R) and two catalytic (C) subunits. The binding of cAMP to allosteric sites on the R subunits produces a conformational change in the R_2_C_2_ complex, causing it to release the C subunits for their function.

The R subunits of the *S. cerevisiae* PKA complex are identical and constituted by Bcy1 while the C subunits vary (Fig. 3*A*). Any two of three different C subunits, Tpk1, Tpk2 and Tpk3, possessing slightly different roles and varying expressions under specific growth conditions, may be part of the R_2_C_2_ complex (102, 103, 104). Their partial redundancy ensures that the expression of just one of them is sufficient for viability (105). But their differential expressions and regulations ensure that the organism is able to respond appropriately to changing environmental conditions (103, 106). Loss of Tpk2 makes *S. cerevisiae* cells unable to undergo yeast to pseudohyphae transitions, while loss of Tpk1 or Tpk3 hyperactivates this transition (104). The two main downstream targets of Tpk2 are the transcription factors, Sfl1 and Flo8. Phosphorylation by Tpk2 inactivates Sfl1 and prevents binding to the promoter of *FLO11*, a gene encoding a GPI-anchored protein required for pseudohyphae production as well as invasive growth. In contrast, phosphorylation of Flo8 by Tpk2 enables it to bind to the promoter of *FLO11* and activate transcription.

The R subunits of *C. albicans* PKA too are encoded by a single gene, *BCY1*, whose homozygous null strains are viable (107). But the C subunit exists in only two isoforms, Tpk1 and Tpk2, which are mutually redundant for vegetative growth but exhibit slightly different substrate specificities and so respond to different environmental stimuli (Fig. 3*B*) (108). Tpk1 is crucial for growth on solid media at 37 °C, but for agar invasion Tpk2 is required. The latter almost exclusively also controls filamentation in liquid Spider medium (containing mannitol) or serum. In *C. albicans* Efg1 is the main downstream activator and Nrg1 the major repressor of hyphae-specific genes downstream to the cAMP-PKA pathway.

#### Cyr1 and cAMP production

**Figure 4:**
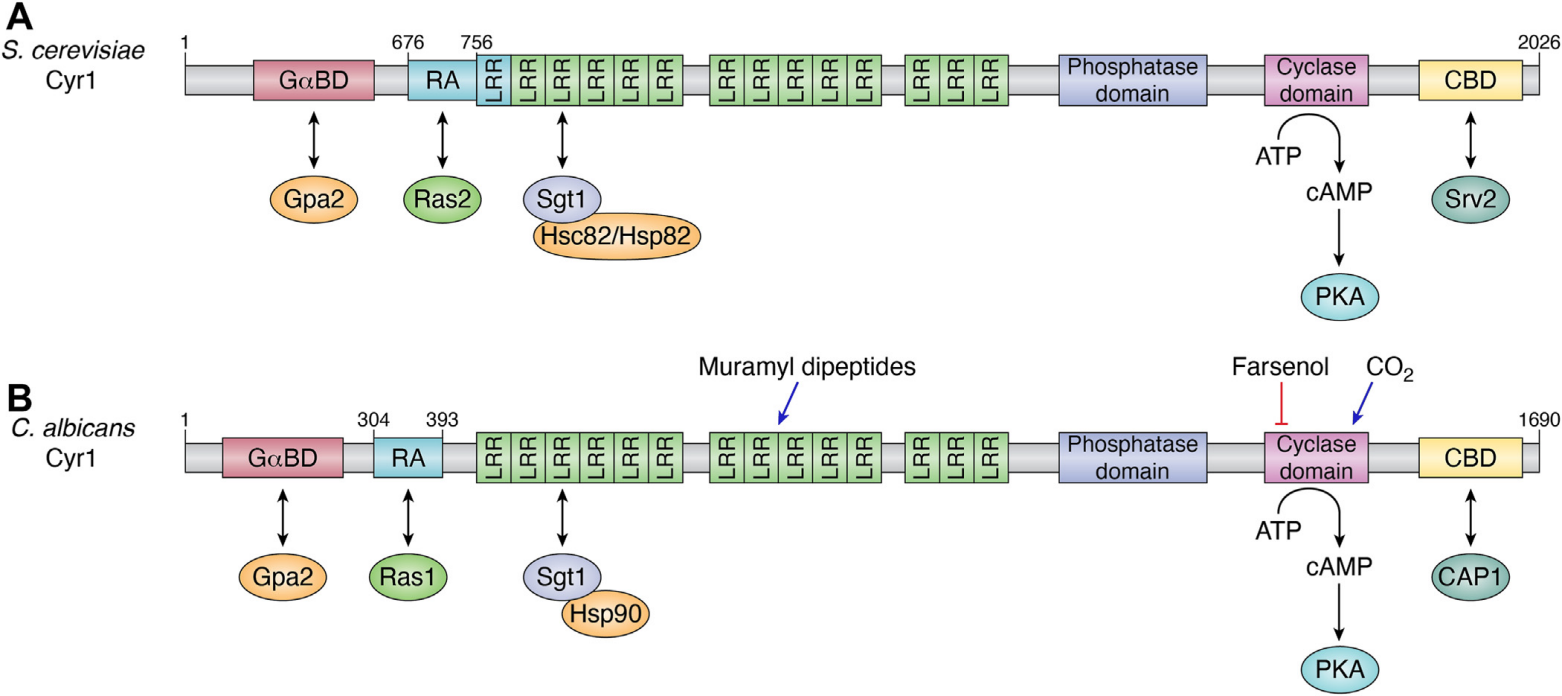
**Domain organization of Cyr1 in *Saccharomyces cerevisiae* and *Candida albicans*.** A schematic representation of the domains of Cyr1 from (*A*) *S. cerevisiae* and (*B*) *C. albicans*. Cyr1 is the adenylyl cyclase that converts ATP to cAMP and thereby activates PKA. This enzymatic activity primarily requires the cyclase domain of Cyr1. The other domains act as sensors of different proteins/signals to regulate cAMP production by the cyclase domain. The Gα-binding domain (GαBD) binds to the α subunit (Gpa2) of a G-protein; Ras association (RA) domain interacts with Ras proteins; the leucine-rich repeat (LRR) domain interacts with Hsp90 proteins *via* their co-chaperone, Sgt1; and the cyclase associated protein 1 (CAP1) binding domain (CBD) interacts with Srv2/CAP1 protein. The role of the protein phosphatase 2C (PP2C) domain is not known. In *C. albicans*, the LRR domain is activated by bacterial peptidoglycans and muramyl dipeptides (shown by *bluearrow*). Its cyclase domain can be activated independently by CO_2_ (shown by *bluearrow*) and repressed by farnesol (represented by *red flatheaded arrow*). Hyperactive mutants of *C. albicans* Cyr1 (Cyr1^E1541K^) can simultaneously bind Ras1 and CAP1. Double headed arrows represent protein–protein interactions.

How is the cAMP required for PKA-dependent signaling produced by the fungal cell? At the heart of this system is the large multi-domain enzyme, adenylyl cyclase (Cyr1 or Cdc35), which produces cAMP from ATP (109, 110). The *S. cerevisiae* and *C. albicans* adenylyl cyclases may be viewed as possessing at least six major domains through which multiple signals are integrated into the cAMP-PKA signaling response (Fig. 4).

They may be listed as follows:

i. A N-terminal Gα-binding domain (GαBD) that binds to the α subunit (Gpa2) of a G-protein (111).
ii. A Ras binding/association (RA) domain. It corresponds to the regions 676 to 756 amino acids in *S. cerevisiae* and 304 to 393 in *C. albicans* (112, 113). Two specific mutations in this domain, at K338A or L349A, can individually block Ras binding to *C. albicans* Cyr1.
iii. A large leucine rich repeats (LRR) domain with multiple 23-residue LRR motifs. In *S. cerevisiae* this domain is contiguous with the RA domain which too contains LRR motifs (112). In *C. albicans* the LRR domain is distinct from the RA domain and is a sensor of bacterial peptidoglycans and muramyl dipeptides (113, 114). In both organisms the LRR domain also interacts with their respective Hsp90 proteins *via* the co-chaperone, Sgt1 (115, 116).
iv. A putative protein phosphatase 2C (PP2C) domain whose function is not yet elucidated (113).
v. The crucial Cyclase domain, which converts ATP to cAMP. In *C. albicans* this domain is also involved in CO_2_ and farnesol sensing (117, 118).
vi. A C-terminal cyclase associated protein 1 (CAP1) binding domain with three conserved motifs of which motifs II and III are required for CAP1 binding (117). In order to maintain its activation status and control cAMP levels, Cyr1 must bind to CAP1, which in turn must bind G-actin (118, 119).

Excess cAMP, not used in PKA activation, can have detrimental effects on the cell. So it is hydrolyzed by the action of two phosphodiesterases, Pde1 and Pde2 (Fig. 3, *A* and *B*). The primary role for regulating the basal cAMP levels falls on the high affinity phosphodiesterase, Pde2 (120). But in the presence of glucose and a transient rise in cAMP levels the low affinity enzyme, Pde1, comes into play (121, 122). PKA itself phosphorylates Pde1 to activate it, making Pde1 crucial to feedback inhibition of cAMP accumulation (123, 124).

Covered in the next few sections are the different mechanisms by which Cyr1 is activated by proteins or signaling molecules that associate with its different domains.

#### Ras proteins and Ras-dependent activation of Cyr1

**Figure 5:**
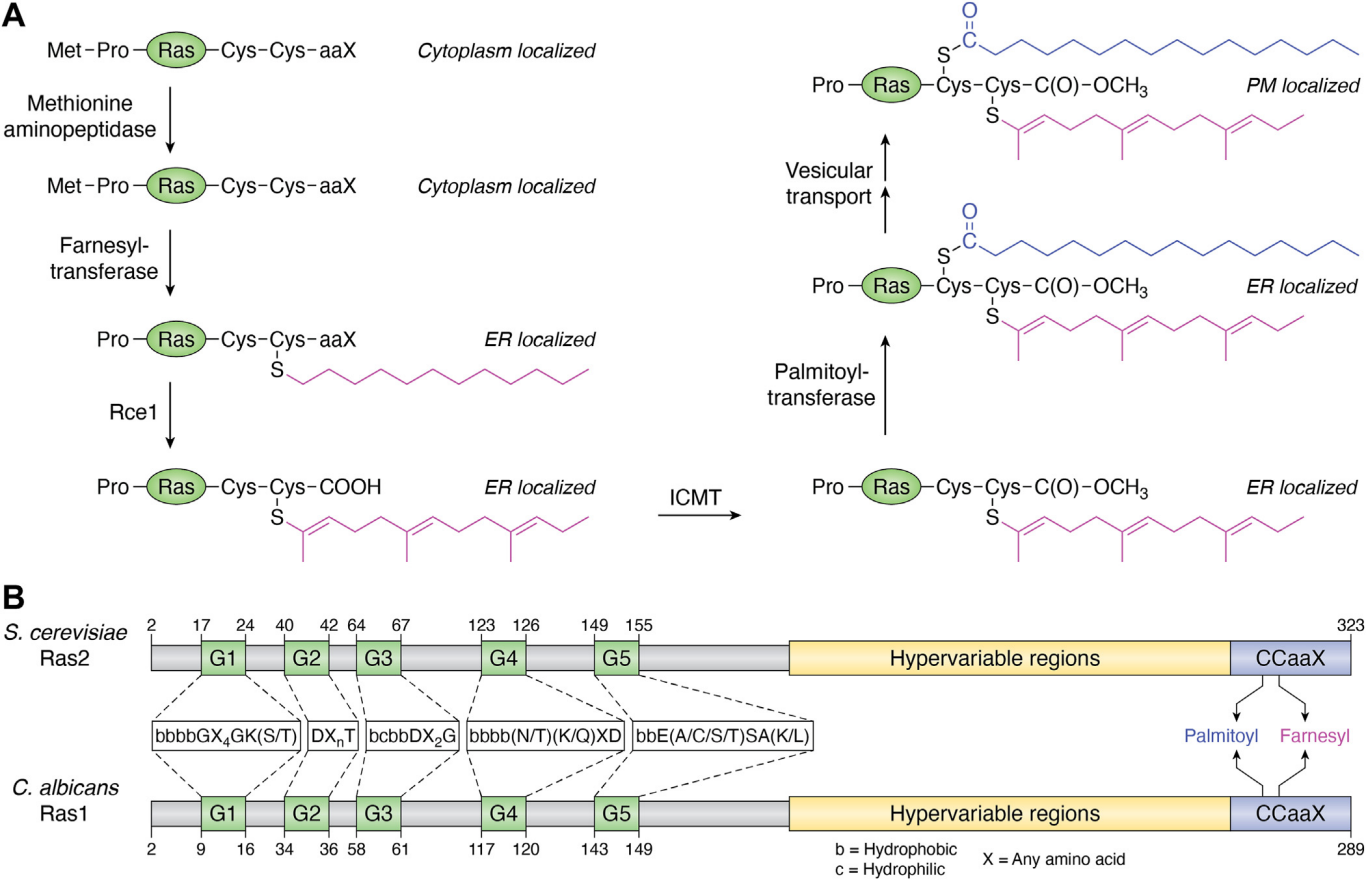
**Ras proteins of *Saccharomyces cerevisiae* and *Candida albicans*.***A*, post-translational modifications of Ras proteins and their subcellular localization. Ras proteins are produced in the cytoplasm. The N-terminal Met is removed almost immediately by methionine aminopeptidase and a farnesyl chain is added to the Cys residue of the C-terminal CAAX (C: Cys, A: aliphatic amino acids, X: the C-terminal amino acid) domain by a farnesyltransferase. The AAX motif is then proteolytically cleaved by an endopeptidase, Rce1, and the new C-terminal -COOH generated is converted to an ester by the action of an isoprenylcysteine methyltransferase (ICMT). Finally, a palmitoyltransferase enzyme complex adds a palmitoyl chain to an adjacent Cys and the protein is transported to the PM. *B*, domain organization of mature ScRas2 and CaRas1. Mature Ras proteins contain five well-conserved G boxes at their N-terminus that are required for GTP binding. The G1 and G2 motifs bind Mg^+2^-bound β and γ P_i_, respectively. The crucial Gln required for GTPase activity is located in the G3 motif. G4 and G5 motifs bind guanine and the ribose sugar. Nucleotide exchange after GEF binding induces large conformational changes in switch I and switch II regions. ScRas2/CaRas1 also have hypervariable regions (HVR) whose sequences are poorly conserved. The C-terminal CAAX domain and the residues on which farnesylation and palmitoylation occur are specifically shown.

Ras proteins are small monomeric GTPases upstream of Cyr1. Unlike in the mammalian system, yeast Ras proteins are not transported to the PM *via* the Golgi (125). They are synthesized in the cytoplasm and undergo a series of sequential post-translational modifications (126, 127, 128, 129, 130, 131, 132), which are summed up in Figure 5*A*. Cytoplasmic Ras proteins becomes ER associated only upon farnesylation of the conserved Cys at their C-terminal CAAX motifs and associate with the PM only after they are additionally palmitoylated at an adjacent Cys residue. It is presumed that the transport from the ER to the PM is *via* vesicles, although the exact mechanism is not known. Thus, depending on its modification status, *S. cerevisiae* Ras2 can be found in the cytoplasm, ER or PM (Fig. 5*A*). It may also be found in the nucleus in wild type cells grown in glucose or localize to the mitochondria under specific conditions (133, 134).

GTP-binding by all Ras proteins requires the conserved N-terminal G domain made of five conserved G-boxes (G1 to G5) and two conserved ‘switch’ regions (Fig. 5*B*) (135). This is followed by a C-terminal region with much poorer degree of conservation, called the hypervariable region (HVR) which might explain the differences in how the Ras proteins interact with their downstream effectors and how these may be regulated in different systems.

In their active states Ras proteins may interact with one or more effectors, thereby regulating multiple cellular pathways. They have poor intrinsic GTPase activity and require the presence of additional proteins, GTPase-activating proteins (GAPs) and guanine nucleotide exchange factors (GEFs), to switch between their active and inactive states as shown in Figure 3, *A* and *B*. GAPs directly participate in the hydrolysis of GTP and release inorganic phosphate (P_i_) by providing additional residues inside the active site *via* a conserved ‘arginine finger’ (136). The resultant GDP remains bound to Ras until its dissociation and the simultaneous binding of another GTP molecule into the active site, which is stimulated by the binding of a GEF to the Ras protein (137, 138).

*S. cerevisiae* has two functionally homologous Ras proteins, Ras1 and Ras2, with differential expressions in different growth conditions that ensure that at least one of them is normally available in a given situation (139, 140). It has one GEF (Cdc25) and two functionally homologous GAPs (Ira1 and Ira2), that participate in the activation-inactivation cycle of both its Ras proteins (Fig. 3*A*). The HVR regions of Ras1 and Ras2 show some differences due to which there appear to be some differences in the manner in which they interact with Cyr1. This probably explains why recombinant Ras2 purified from bacterial cells shows greater stimulation of cAMP production by Cyr1 in reconstitution experiments than Ras1 (141). The interaction of Cyr1 with Ras2-GTP is modulated by GAPs, Ira1 or Ira2, and their ability to stimulate GTP hydrolysis. A Gly19Val mutation in Ras2 can prevent this interaction and keep it in a GTP-bound, constitutively activated state (142). Cyr1 has a second, lower affinity, GTP-dependent binding site for Ras2, which is only created when CAP1 binds to Cyr1 (109, 143). This binding specifically requires Ras2 to be farnesylated and causes significant stimulation of cAMP production.

Intracellular glucose sensing and its conversion to fructose 1,6-bis-phosphate (Fru1,6bisP) can also activate Ras-dependent cAMP production in *S. cerevisiae* (Fig. 3A)*.* Fru1,6bisP is sensed by the N-terminal domain of Cdc25, which then goes on to switch Ras2 from its inactive to active form, thereby enabling activation of Cyr1 (144, 145, 146).

*C. albicans* also has two Ras proteins: CaRas1, which is homologous to both the *S. cerevisiae* Ras proteins; and CaRas2, which is distinct, shows poor homology to CaRas1 as well as to both the *S. cerevisiae* Ras proteins, and plays an antagonistic role to CaRas1 in cAMP production (147). CaRas2 does not seem to bind to Cyr1 (113, 147). It is, therefore, unclear why complete deletion of *CaRAS2* in *Caras1Δ/Δ* cells aggravates filamentation defects of the strain. But what is clear is that CaRas1 alone can rescue a *ras2Δ* strain as well as functionally replace Ras2 in the cAMP/PKA signaling pathway of *S. cerevisiae* (147). CaRas1 is also post-translationally farnesylated and palmitoylated at Cys^288^ and Cys^287^, respectively, resulting in its localization to the ER and the PM (Fig. 5*A*). Once at the PM it can participate in cAMP-PKA signaling or get inactivated by regulated proteolysis (when farnesol is present) (148). This latter process is not reported in *S. cerevisiae*.

As in *S. cerevisiae*, *C. albicans* Cdc25 is required for the activation of its Cyr1 by glucose (Fig. 3*B*) (149). Since it is a functional homolog of *S. cerevisiae* Cdc25 (150), it is possible that the mechanism by which this occurs is conserved in the two organisms. *C. albicans* possesses a single GAP, Ira2. Based on sequence and functional conservation, it appears that CaRas1^G13V^ cannot interact with the GAP, Ira2, resulting in a constitutively activated Ras protein.

#### Role of Hsp90 in Ras-Cyr1-cAMP-PKA signaling

The next major difference in the Cyr1-cAMP-PKA signaling of the two organisms involves how the two pathways are regulated by their respective Hsp90 proteins. Hsp90 proteins are a family of highly conserved and essential cytoplasmic heat-shock proteins that act as molecular chaperones in prokaryotes and eukaryotes. Their expression is regulated by the heat-shock transcription factor, Hsf1, in both organisms (151). *S. cerevisiae* expresses two isoforms of Hsp90, the constitutively expressed Hsc82, and the heat-inducible Hsp82. The two proteins are roughly 97% identical in their amino acid sequence and share overlapping interactomes with some differences (152, 153). *C. albicans* expresses a single isoform of Hsp90. Under physiological conditions, *C. albicans* Hsp90 acts on a set of client proteins to stabilize and fold them (154). Hence, it participates in several cell signaling events to regulate crucial attributes of the pathogen such as cell viability, stress response, drug sensitivity, morphogenesis and virulence.

As mentioned earlier, in both *S. cerevisiae* as well as *C. albicans*, the LRR domain of Cyr1 interacts with Hsp90 proteins *via* the co-chaperone, Sgt1 (Fig. 4) (115, 155). However, the consequences of these interactions are very different. In *S. cerevisiae* mutations that interfere with the association between Hsc82 (or Hsp82) and Sgt1 also result in a downregulation of Ras-dependent cAMP production by Cyr1 (152). In other words, Hsc82-Sgt1 activates cAMP production by Cyr1 in a Ras-dependent manner (Fig. 3*A*). In thermal stress, Hsp82 expression increases and equals that of Hsc82 (153). This leads to greater activation of the cAMP-PKA pathway and produces hyperactive Ras phenotypes. *C. albicans* Hsp90-Sgt1, on the other hand, is an inhibitor of Ras1-Cyr1 association (Fig. 3*B*) and limits cAMP production (116, 155, 156). When the cell is subjected to thermal stress, Hsp90 gets diverted to the refolding of heat-unfolded/misfolded proteins which relieves the inhibition on CaRas1-Cyr1 interaction and promotes cAMP production, resulting in filamentation.

#### CAP1 and its role in activation of Cyr1-cAMP-PKA signaling

Although CAP1 proteins are conserved in all eukaryotes, they do not play a universal role in activation of the cAMP-PKA pathway. Their most conserved function is in actin dynamics, where they participate in many processes including in the stabilization of the barbed end of polymerized actin (F-actin) to inhibit its growth and promote its depolymerization (157, 158).

In *S. cerevisiae* and *C. albicans*, the N-terminal end of CAP1 binds to Cyr1 at its C-terminal end. The C-terminal end of Srv2/CAP1 proteins bind to monomeric G-actin (Fig. 3, *A* and *B*). As mentioned earlier, CAP1 binding to Cyr1 produces a second Ras binding site that stimulates cAMP production in *S. cerevisiae* (143). In fact, CAP1 was first identified in *S, cerevisiae* as the product of the suppressor of Ras2V19 (*SRV2*) gene (159). Mutants deficient in *SRV2* expression are unable to accumulate excessive cAMP even when expressing the constitutively active protein, Ras2^V19^, and do not exhibit hyperactive Ras phenotypes.

In *C. albicans* Cyr1-CAP1-G-actin appear to form a trimolecular signaling platform that can be activated by various signals in a Ras-independent and actin-dependent manner (119, 160, 161). It is even possible to speculate that this is the complex responsible for the production of basal levels of cAMP in the cell. This would also explain why a double homozygous null strain, *Caras1ΔΔ/Caras2ΔΔ*, is viable.

Isolated Cyr1-CAP1-G-actin can be activated by serum or muramyl dipeptides to produce cAMP *in vitro* (119). The same can be blocked in wild type cells by inhibitors of actin polymerization, cytochalasin D and latrunculin B, but not in cells expressing a mutant of CAP1 lacking the G-actin binding site. Mutations in CAP1 that inhibit G-actin binding also partially block cAMP production, suggesting that this interaction is required for the full activation of Cyr1 (119). Although its mechanism is unclear, it appears that actin polymerization may be an inherent feature of filamentation *via* Cyr1-CAP1-G-actin. It is possible that G-actin recruitment for Cyr1 activation ensures that it is unavailable for F-actin depolymerization activities, thereby enabling CW remodeling and hyphal elongation to accompany cAMP production.

Does CaCyr1 simultaneously bind CAP1-G-actin on the one hand and CaRas1 on the other? The preceding discussion suggests that they do not necessarily always work together, although such an assembly has been speculated under specific conditions. For example, a hyperactive mutant Cyr1^E1541K^ requires both CaRas1 and CAP1 for its phenotypes (filamentation as well as riboflavin synthesis) (117). Complexes of CaRas1-Cyr1 (113) and Cyr1-CAP1-G-actin (as mentioned above) are readily isolated but attempts to isolate a CaRas1-Cyr1-CAP1-G-actin complex have not been successful. The inhibitory effect of Hsp90 on the association of CaRas1 with Cyr1 (Fig. 3*B*) probably limits the chances of isolating such a complex under normal conditions. It is also possible that these associations are transient and occur only in intact cells during the signaling event.

Some clues are available, however, from studies on the diffusion dynamics of N-terminally mRFP-tagged CaRas1 in wild type cells *versus* cells that overexpress either CaRas1 (*CaRAS1oe* strain) or a constitutively activated variant, CaRas1^G13V^ (*CaRAS1*^*G13V*^*oe* strain) which cannot interact with Ira1 to return to its inactive state (162). Hyperfilamentation in Spider medium is exhibited by both the overexpression strains. However, in the *CaRAS1oe* strain the diffusion dynamics of CaRas1 is indistinguishable from that observed in wild type cells, while in the *CaRAS1*^*G13V*^*oe* strain it is significantly slower, as though CaRas1 is part of a much larger complex. Similar slow (or ‘hyperactivated’) CaRas1 dynamics is observed in the hyperfilamentous cells of a conditional null strain of *HSP90*. ‘Hyperactivated’ dynamics for CaRas1 is also observed in wild type cells treated with geldanamycin, a Hsp90 inhibitor. Conversely, the dynamics of CaRas1 becomes comparable to that in the wild type strain (‘normal’ dynamics) when cells of the conditional null strain of *HSP90* are treated with tamoxifen, a Hsp90 activator. ‘Hyperactivated’ CaRas1 dynamics is also observed in wild type cells when F-actin is stabilized with jasplakinolide. On the other hand, actin depolymerization by cytochalasin D can restore to ‘normal’ the dynamics of CaRas1 in the conditional null strain of *HSP90* and in cells of *CaRAS1*^*G13V*^*oe*. Taken together, these results too suggest that, 1) actin polymerization is a feature of ‘hyperactivated’ CaRas1 and, 2) CaRas1-Cyr1-CAP1-G-actin signaling platforms may be formed in *C. albicans* cells under special circumstances. We shall return to this in the last section of this review.

#### Other activators/inhibitors of Cyr1-cAMP-PKA signaling

*S. cerevisiae* Cyr1 can be stimulated in other ways too that do not directly involve a role for Ras. For example, it can be activated by GTP-bound Gpa2 *via* its N-terminal Gα binding domain (Figs. 3*A* and 4*A*) (163, 164). Gpa2 in turn is activated by Gpr1, a G-protein coupled receptor and sensor of extracellular glucose, which acts like a GEF for Gpa2 (Fig. 3*A*) (165, 166). *S. cerevisiae* Gpr1 has a relatively weak affinity for glucose and a higher affinity for sucrose (166) which ensures that the organism switches from respirative to fermentative metabolism only at high glucose concentrations.

*C. albicans* possesses a Gpr1-Gpa2 signaling system homologous to that in *S. cerevisiae*. However, CaGpr1 does not sense glucose. Instead it senses either lactose or methionine or both (Fig. 3*B*) (149, 167, 168). As in *S. cerevisiae*, CaCyr1 can be directly activated by CaGpa2 binding to its N-terminal Gα binding domain (Figs. 3*B* and 4*B*). In addition, it can be activated by muramyl dipeptides and bacterial peptidoglycans *via* its LRR domain (Fig. 4*B*), unlike in *S. cerevisiae*. More importantly, from an infection and virulence perspective, the cyclase domain of CaCyr1 is a HCO_3_^-^/CO_2_ sensor that is activated to produce cAMP, independent of CaRas1 as well as CAP1 (Figs. 3*B* and 4*B*) (117, 118). This function is absent in *S. cerevisiae* Cyr1. Quorum-sensing molecules like farnesol can also bind to this domain and block cAMP production (Figs. 3*B* and 4*B*) (117). In cells where Cyr1 is inhibited by farnesol, CaRas1 undergoes regulated proteolysis to be converted into a soluble cleavage product that is incapable of interacting with CaCyr1 (148). This again is a feature unique to *C. albicans*. Thus, besides being activated by CaRas1, *C. albicans* Cyr1 seems to be specially adapted to transduce many more signals and integrate them into the cAMP/PKA response for hyphal morphogenesis than its *S. cerevisiae* counterpart.

As previously mentioned, filamentation in *S. cerevisiae* and *C. albicans* is also induced under nitrogen starvation conditions. Gpa2 becomes phosphorylated and PM-localized for direct activation of Cyr1 under such conditions (111). Three ammonium permeases are reported in *S. cerevisiae* (Mep1/2/3), and two in *C. albicans* (Mep1/2). Of these, Mep2 is the transceptor, functioning both as a transporter as well as signal transducer (169, 170). It was previously proposed that the C-terminal domain of CaMep2 was involved in transducing the nitrogen starvation signal to CaRas1 and cells of *Caras1* were unable to filament in low nitrogen conditions even when hyperactive Mep2 was overexpressed (170) but a more recent study suggests that homologs from both organisms may function by making the cytosol more alkaline (171). It is unclear how CaRas1 could be involved, if at all. The well-studied Rim21-Rim101 pathway is also known to contribute to alkaline pH-induced filamentation, but in a CaRas1-cAMP-PKA-independent manner (172). Under low pH conditions, the cAMP-PKA signaling is also inhibited *via* Cyr1, which is negatively regulated by acidic pH, but this too is a Ras-independent process (173). *N*-acetylglucosamine (GlcNAc) is another well-known hyphae inducer that was previously believed to induce filamentation in a cAMP-dependent manner in *C. albicans*. While it does seem to require a basal level of cAMP production, recent studies indicate that GlcNAc-dependent hyphal morphogenesis may not otherwise require the Cyr1-cAMP-PKA pathway (174).

## Crosstalk between GPI biosynthesis and cAMP/PKA signaling

How does GPI biosynthesis cross-talk with filamentation signaling in *S. cerevisiae* and *C. albicans*? All experimental evidence available so far indicates that the two pathways intersect *via* the first step of GPI biosynthesis.

### Mutual interaction and a probable model for the cross-talk between Ras and GPI-GnT in *S. cerevisiae*

Eri1 and Gpi2 subunits of the GPI-GnT co-precipitate with one another as well as with GTP-bound Ras2 in an effector loop-dependent manner from ER fractions of *S. cerevisiae* cell lysates (12, 175). It is not clear whether one or both subunits directly bind to Ras2 or whether the interaction is mediated by any of the other subunits. Sobering *et al.* (12), also cite unpublished results to suggest that Eri1 *per se* is not required for the association of Ras2 with the GPI-GnT (12). This hints at a possible bridging interaction, that brings together Eri1 and Ras2. Could Gpi2 be the main interacting subunit? Bimolecular fluorescence complementation assays for the interaction of Ras2 with Gpi2 do hint at such a possibility (unpublished data, Komath lab). Temperature sensitive *gpi1*, *gpi15*, and *gpi19*, strains show hyperactive Ras phenotypes such as greater heat shock sensitivity, hyperfilamentation and/or increased invasive growth, suggesting that any one of these too could be involved in the interaction. No matter the actual interacting partner, it appears that the GPI-GnT as a whole is an inhibitor of GTP-dependent Ras-cAMP-PKA signaling in *S. cerevisiae*. Thus, the GPI-GnT may be viewed as a “Ras effector”, a protein that interacts with the active form of Ras (12).

The above study also shows that the interaction between Ras and the GPI-GnT improves with Ras2^G19V^, the GTP-bound constitutively activated form of Ras2, and reduces when a mutation to inhibit effector association (either T42A or N45) is introduced into Ras2^G19V^ (12). Interestingly, the GlcNAc transferase activity of the GPI-GnT in a strain expressing Ras2^G19V^ is significantly reduced. On the other hand, *ras2Δ* shows significantly enhanced GPI-GnT activity. Thus, Ras2 is an inhibitor of the GPI-GnT.

**Figure 6:**
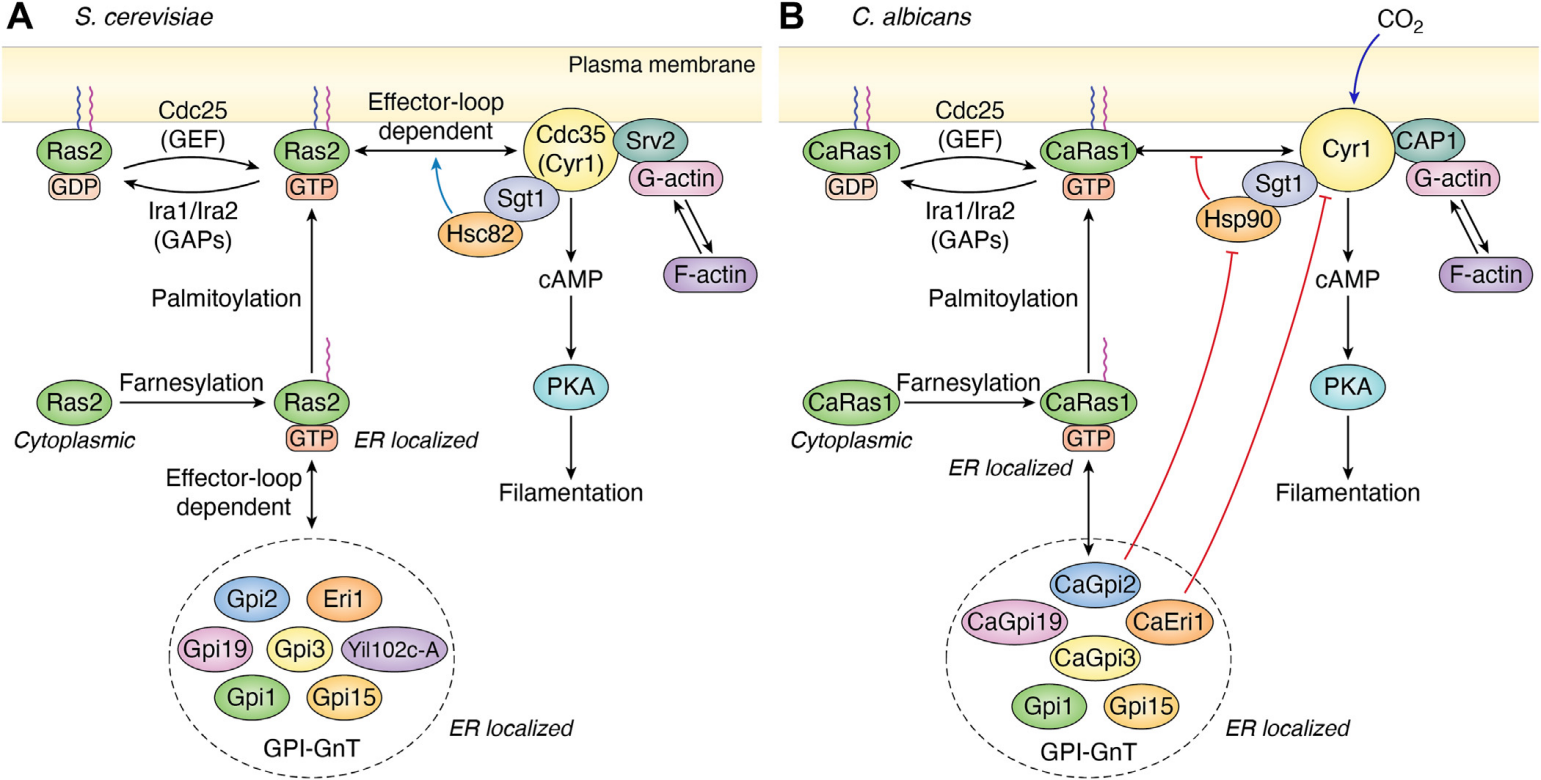
**Cross-talk of GPI-GnT with Cyr1-cAMP-PKA pathway in *Saccharomyces cerevisiae* and *Candida albicans*.** Shown in the figure are probable models of the cross-talk in (*A*) *S. cerevisiae* and (*B*) *C. albicans*. Ras proteins interact with the GPI-GnT at the ER during their transit to the PM. How this transition occurs is shown in Figure 5. This requires active Ras and, at least in *S. cerevisiae*, the effector loop. The interaction leads to inhibition of the GPI-GnT in *S. cerevisiae* and its activation in *C. albicans*. In *C. albicans*, only the Gpi2 subunit physically interacts with Ras. Perhaps the same subunit interacts with Ras in *S. cerevisiae*, but no clear-cut evidence exists for this. The GPI-GnT in *S. cerevisiae* inhibits the Cyr1-cAMP-PKA signaling. One possible model would be to assume that when bound to the GPI-GnT in the ER, the molecules of Ras available at the PM to activate cAMP production drop. This leads to the inhibition of filamentation. Downregulation of any of the GPI-GnT subunits releases Ras and activates filamentation. In *C. albicans*, overexpression of Gpi2 inhibits Hsp90, whose downregulation enables better interaction of Ras with Cyr1 and activates filamentation. If such an effect were to operate in *S. cerevisiae*, it would lead to inhibition of filamentation, since Hsc82 promotes the interaction of Ras with Cyr1 in this organism. But evidence in support of such a model is lacking. Filamentation in *C. albicans* is also activated by the direct binding of CO_2_ to the cyclase domain of Cyr1, independent of Ras. The Eri1 subunit of the GPI-GnT inhibits this process. *Double headed arrows* represent protein–protein interactions, *red flatheaded arrows* represent inhibition, and *blue arrows* represents activation.

Taken together it may be concluded that Ras signaling and GPI biosynthesis are mutually inhibitory in *S. cerevisiae*. One simple hypothesis that would explain these results would be to assume that the interaction of Ras2 with the GPI-GnT (perhaps *via* its Gpi2 subunit), inhibits the latter at the ER. It also concomitantly retards the arrival of Ras2 at the PM, thereby limiting its availability for Cyr1-cAMP-PKA activation (Fig. 6*A*). Thus, both GPI biosynthesis and filamentation are simultaneously inhibited. It is possible that deficiency in any of the GPI-GnT subunits destabilizes the other subunits and the GPI-GnT assembly, resulting in inefficient interaction of Gpi2 with Ras2. This allows Ras2 to localize to the PM in larger amounts, producing hyperfilamentation phenotypes. Similarly, when Ras2 is downregulated, its interaction with the GPI-GnT is reduced and inhibition of the GlcNAc transferase activity is abrogated. Overexpression of Ras2 would further inhibit GPI-GnT activity, but hyperactivate cAMP-PKA signaling.

### The interaction between Ras and the GPI-GnT in *C. albicans* and a probable model for the cross-talk

Several lines of evidence suggest that it is possible to decouple GPI-biosynthesis from cAMP-PKA dependent filamentation in *C. albicans*. Yet, in the wild type cell, each pathway appears to have an influence on the other.

#### Activation of GPI-GnT by Ras

CaRas1 is an activator of the GPI-GnT in *C. albicans* (22). ER-localized or cytoplasmic CaRas1 better activates GPI-GnT than its PM-localized form. The constitutively active form, CaRas^G13V^, is the preferred activator. Affinity pull-down experiments bring down CaRas1 and CaEri1 with CaGpi2 suggesting that they are part of the same complex (14). Confocal microscopy imaging suggests that the three proteins colocalize at the ER. Acceptor-photobleaching fluorescence resonance energy transfer studies suggest that CaRas1 and CaGpi2 proteins are probably close enough for a direct physical interaction, as are CaEri1 and CaGpi2 (14, 22). But this is not the case for CaEri1 and CaRas1. Thus, it may be inferred that the physical interaction of GTP-bound CaRas1 with CaGpi2 is specific, occurs in the ER, and does not simultaneously involve CaEri1.

*S. cerevisiae* Ras2 cannot replace CaRas1 in GPI-GnT activation even though it rescues growth, activates cAMP production, and promotes filamentation (176). Perhaps the poorly conserved HVR region of the two Ras proteins are responsible for these differences. The domains of Ras2 through which it interacts with the well conserved GEF, GAP and Cyr1 proteins of *C. albicans* are located in the highly conserved N-terminal half of the protein, which probably explains its ability to functionally complement CaRas1 in the cAMP-PKA pathway at the PM. Alternatively, these differences arise due to the poor conservation of the GPI-GnT subunit(s) with which the Ras protein interacts. As explained earlier, the Gpi2 subunits of *C. albicans* and *S. cerevisiae* do not complement one another (27).

#### Activation of filamentation by the GPI-GnT

Filamentation in *C. albicans* involves the Ras-dependent as well as Ras-independent Cyr1-cAMP-PKA pathway. The GPI-GnT influences both.

##### Activation of filamentation *via* the Ras-dependent Cyr1-cAMP-PKA pathway

Mutant strains of individual GPI-GnT subunits in *C. albicans* do not show the same hyphal morphogenesis phenotype. In media that activate Ras-dependent cAMP-PKA signaling (such as YEPD, Spider, or serum), heterozygous strains of *CaGPI1*, *CaGPI2*, *CaGPI3* and *CaGPI15* have reduced cAMP-PKA activity and are hypofilamentous, while those of *CaGPI19* and *CaERI1* have higher cAMP-PKA levels and are hyperfilamentous. Each of these phenotypes correlates with the level of CaGpi2 expressed in the strains (22). The hypofilamentous strains have lower CaGpi2 levels while the hyperfilamentous ones have increased CaGpi2 levels (CaGpi2 levels are altered due to unique inter-subunit transcriptional regulations, as explained earlier). Overexpressing *CaGPI2* in heterozygous *CaGPI1*, *CaGPI3* and *CaGPI15* strains or downregulating it in the heterozygous *CaGPI19* and *CaERI1* strains, restores the levels of cAMP and the extent of filamentation to wild type levels in Spider medium. Thus, filamentation in the Ras-dependent medium is controlled by CaGpi2 in all the GPI-GnT mutant strains. The enzymatic activity of the GPI-GnT is not restored in any of the strains where CaGpi2 is overexpressed or downregulated, suggesting that the role of CaGpi2 in filamentation is independent of its role in GPI-GnT activity.

Overexpressing *CaRAS1* in a heterozygous *CaGPI2* strain reverses its filamentation defect while overexpressing *CaGPI2* in a homozygous null strain of *CaRAS1* does not produce any hyphae in the same media, proving that CaRas1 is downstream of CaGpi2 in the Ras-dependent cAMP-PKA signaling pathway (30). How does CaGpi2 control Ras-dependent filamentation? *CaGPI2* overexpression causes Hsp90 downregulation and reduces Hsp90 activity. This in turn should relieve the Hsp90-mediated inhibition of CaRas1-Cyr1 interaction (Fig. 6*B*) and enable increased cAMP production. As expected, the wild type strain overexpressing *CaGPI2* is hyperfilamentous in Spider medium or YEPD at 30 °C, has high levels of F-actin even in yeast cells, and possesses ‘hyperactivated’ CaRas1 as tracked by FCS (22, 162).

Hypothetically speaking, a similar mechanism could work in *S. cerevisiae* as well, where Ras interacts with and inhibits the GPI-GnT at the ER and Gpi2 independently inhibits Hsc82 (or Hsp82). Since the heat shock proteins are activators of Cyr1-cAMP-PKA signaling, this could well explain the phenotypes observed. However, no evidence exists yet to suggest that GPI-GnT activity and filamentation in this organism is, or can be, decoupled.

##### Activation of filamentation *via* the Ras-independent pathway

Yeast cells of heterozygous and conditional null strains of *CaERI1* have high levels of F-actin and appear to be already primed for filamentation (14). When grown in HCO_3_^-^/CO_2_ at 37 °C, they show longer and increased numbers of hyphae than their wild type controls. Even in the homozygous null strain, *Caras1ΔΔ*, disrupting one allele of *CaERI1* produces better filamentation in HCO_3_^-^/CO_2_ relative to the parent strain. Two inferences may be drawn from these results: 1) CaRas1 is not required for filamentation in HCO_3_^-^/CO_2_ (as also mentioned previously), and 2) CaEri1 is a Ras-independent inhibitor of cAMP/PKA signaling for hyphal morphogenesis. When one allele of *CaERI1* is disrupted in heterozygous strains of *CaGPI1*, *CaGPI2*, *CaGPI3*, *CaGPI15* and *CaGPI19*, the double heterozygous strains show better filamentation in HCO_3_^-^/CO_2_ than the parent strains. Thus, among the GPI-GnT subunits, CaEri1 is the main inhibitor of Cyr1 activation *via* HCO_3_^-^/CO_2_. The transcriptional cross-talk is worth noting. *CaGPI2* is overexpressed in the heterozygous *CaERI1* strain, while overexpression of *CaGPI2* in the wild type strain suppresses *CaERI1* expression.

If one allele of *CaRAS1* or *CaGPI2* is disrupted in a heterozygous strain of *CaERI1*, it does produce a small drop in the hyperfilamentation phenotype of the parent strain, which would be attributable to reduced basal levels of cAMP. In other words, the Cyr1-cAMP-PKA pathway in the mutant strain of *CaERI1* continues to simultaneously responding to the Ras-dependent pathway. Taken together with the transcriptional cross-talk and the F-actin levels, it may be inferred that the Cyr1-cAMP-PKA pathway is ‘hyperactivated’ in the *CaERI1* deficient strains, just like it is in the *CaGPI2* overexpression strain.

#### A probable model for cross-talk of GPI-GnT with filamentation in *C. albicans*

Summarizing all the information discussed above permits us to propose a model for how GPI-GnT and filamentation are regulated in *C. albicans* (Fig. 6*B*)*.* The GPI-GnT primarily exists at the ER in this organism as in *S. cerevisiae*. After being synthesized in the cytoplasm, CaRas1 too transits through the ER *en* *route* to the PM. During this phase its active GTP-bound form physically interacts with the CaGpi2 subunit alone of the GPI-GnT. This results in activation of the GPI-GnT.

The effect of the GPI-GnT on filamentation *via* the Cyr1-cAMP-PKA signaling occurs at the PM and is independent of the ER-localized interaction. It also involves at least two different GPI-GnT subunits, CaGpi2 and CaEri1. CaGpi2 activates the Ras-dependent pathway by downregulating Hsp90 and promoting the interaction of GTP-bound CaRas1 with Cyr1. CaEri1 regulates filamentation *via* the Ras-independent pathway, as an inhibitor of Cyr1. Neither of these processes requires any of the other GPI-GnT subunits or an active GPI-GnT enzyme. The mutual transcriptional regulation between *CaGPI2* and *CaERI1* ensures that upregulation of *CaGPI2* downregulates *CaERI1* and *vice versa*, thus, ensuring that irrespective of which arm is activated both arms of the Cyr1-cAMP-PKA signaling pathway are simultaneously engaged, giving rise to ‘hyperactivated’ PKA signaling and hyperfilamentation.

### Summary and perspectives

Both Ras signaling and GPI biosynthesis are unique to eukaryotes. They also happen to be ubiquitously present and reasonably well conserved across eukarya. So much so that mammalian Ras proteins can functionally complement *S. cerevisiae ras2Δ* strain in the Cyr1-cAMP-PKA signaling pathway, making the yeast a useful model for the functional analysis of human Ras proteins (177, 178). This is also true for the GPI biosynthetic pathway. The core of the GPI glycolipid is completely conserved and several mammalian genes can complement yeast GPI mutants (1, 23). Yet crucial differences persist at the structural as well as biosynthetic level, a reflection perhaps of the differences in their evolutionary trajectories.

This Review is an attempt to show how the Ras signaling and GPI biosynthetic pathways cross-talk with one another in two fungal organisms, *S. cerevisiae* and *C. albicans*, both of which have been closely associated with humans throughout history. The former is a non-pathogenic yeast used over the ages for brewing alcohol and producing other fermented products and whose use in modern biology as a model organism has enabled the growth of such diverse areas as genetics, biotechnology, proteomics, functional genomics and chemogenomics. The latter is a commensal that has co-evolved with its host by optimizing strategies for colonizing different niches of the human body and rapidly establishing systemic infections whenever such an opportunity were to present itself (see for example, (179)).

The two organisms also exhibit significant other differences. For instance, both are capable of filamentation, but the morphological switch from yeast cells to filaments in *S. cerevisiae* occurs solely *via* the production of pseudohyphae. In *C. albicans*, on the other hand, both pseudohyphae and true hyphae are produced. These morphological transformations in *C. albicans* are triggered by a more diverse set of cues than in *S. cerevisiae*, many of which are specifically those that it encounters in the human host. The signals that control filamentation in both organisms function *via* a network of pathways. As may be expected, these too are more intricate and complex in *C. albicans* than in *S. cerevisiae.*

Of the many pathways that control filamentation, the Cyr1-cAMP-PKA signaling pathway is common to both organisms. In *S. cerevisiae*, this pathway is Ras-dependent. In *C. albicans* it has both Ras-dependent and Ras-independent arms that can function either separately or in concert in response to distinct cues. *C. albicans* Ras1 and *S. cerevisiae* Ras2 can functionally complement one another to activate Cyr1, indicating that in terms of the Ras-dependent Cyr1-cAMP-PKA signaling the two organisms are quite similar. But this is not quite the full story. Hsp90 proteins regulate the interaction of Ras proteins with Cyr1. In *S. cerevisiae* they promote interaction of Ras with Cyr1, in *C. albicans* they inhibit it. Cyr1 in *C. albicans* has evolved to also independently sense the activator, HCO_3_^-^/CO_2_, or the inhibitor, farnesol, a feature that the *S. cerevisiae* counterpart lacks.

Likewise, at first glance, the GPI biosynthetic pathway of the two organisms appear to be very similar. Both produce similar GPI glycolipids possessing four compulsory Man residues within the carbohydrate linker. All the steps involved in GPI biosynthesis appear to be conserved, with homologs of almost all the *S. cerevisiae* genes being present in *C. albicans*. More importantly, the catalytic subunits of the enzymes catalyzing the various steps appear very well conserved. Yet, there are important differences. For instance, Gpi3, the catalytic subunit of the GPI-GnT, that catalyzes the first biosynthetic step, is inhibited by jawsamycin in *S. cerevisiae* but not in *C. albicans*. Further, inter-subunit transcriptional regulations are absent in *S. cerevisiae* but are a major determinant of drug sensitivities and morphogenesis in *C. albicans*. As explained in this Review, the interaction of the GPI-GnT subunits with the Cyr1-cAMP-PKA pathways too are significantly different in the two organisms. Indeed, they appear to be diametrically opposite in how their activities are controlled by Ras and how they, in turn, control cAMP production for filamentous growth. These differences lie at the heart of how *S. cerevisiae* is able to downregulate its energetically demanding GPI biosynthetic pathway while foraging for nutrients through filamentous growth and how *C. albicans* is able to coactivate hyphal formation with the production of GPI-anchored proteins required for the establishment of invasive infections. Rare cases of opportunistic *S. cerevisiae* infections are characterized by an upregulation of amino acid biosynthesis, DNA damage repair mechanisms and oxidative stress response genes rather than of GPI biosynthesis (180). Expectedly, a genome-wide analysis of genes that were upregulated in an insect model of *S. cerevisiae* infection identified those involved in pseudohyphae production or required for CW integrity but did not simultaneously identify GPI biosynthetic genes or GPI anchored proteins (181). It is also worth emphasizing here that the human host itself exhibits no cross-talk between its Ras signaling and GPI biosynthetic pathways (182).

In conclusion, while commonalities enable us to develop experimental models and generalized theories, exploring species-specific differences are crucial for the development of an organism-specific understanding of processes and to design targeted therapies. It is hoped that this Review, by providing a more nuanced understanding of how apparently similar or conserved pathways operate in two related organisms, will provide its readers with one such starting point.

## Data availability

All the data is contained within the manuscript.

## Conflict of interest

The author declares that they have no conflicts of interest with the contents of this article.
